# The effect of iron availability on transcription of the *Neisseria meningitidis fHbp* gene varies among clonal complexes

**DOI:** 10.1099/mic.0.054957-0

**Published:** 2012-04

**Authors:** Holly Sanders, Carina Brehony, Martin C. J. Maiden, Caroline Vipond, Ian M. Feavers

**Affiliations:** 1 National Institute for Biological Standards and Control, Blanche Lane, Potters Bar, Hertfordshire EN6 3QG, UK; 2 Department of Zoology, University of Oxford, The Tinbergen Building, South Parks Road, Oxford OX1 3PS, UK

## Abstract

Factor H binding protein (fHbp) is a major antigenic component of novel vaccines designed to protect against meningococcal disease. Prediction of the potential coverage of these vaccines is difficult, as fHbp is antigenically variable and levels of expression differ among isolates. Transcriptional regulation of the *fHbp* gene is poorly understood, although evidence suggests that oxygen availability is involved. In this study iron accessibility was found to affect *fHbp* transcription. However, regulation differed among meningococcal clonal complexes (ccs). For the majority of isolates, increased iron concentrations upregulated transcription. This effect was enhanced by the presence of a 181 bp insertion element upstream of *fHbp*, associated with isolates belonging to cc4 and cc5. Conversely, meningococci belonging to cc32 showed iron-repressed control of *fHbp*, as regulation was dominated by cotranscription with the iron-repressed upstream gene *cbbA*. These results highlight the complexity of *fHbp* regulation and demonstrate that control of transcription can vary among genetic lineages.

## Introduction

*Neisseria meningitidis*, the meningococcus, is a major cause of bacterial meningitis and septicaemia. Invasive meningococci typically express one of five capsular polysaccharides, A, B, C, W135 and Y, which are used to type them into serogroups (Jolley *et al.*, 2007). Effective protein–polysaccharide conjugate vaccines are available against serogroup A, C, W135 and Y meningococci (Snape & Pollard, 2005); however, because of its similarity to glycosylated human antigens, the serogroup B polysaccharide is widely regarded as a poor vaccine candidate (Yongye *et al.*, 2008). Instead, the development of a vaccine with the potential to protect against organisms expressing the group B capsule has focussed on subcapsular protein antigens. One such antigen is factor H binding protein (fHbp), a surface-exposed lipoprotein that is a major component of two vaccine candidates: Bexsero (Novartis) and rLP2086 (Pfizer). Both are at an advanced stage of clinical development and have been shown to induce fHbp-specific bactericidal antibodies in humans (Findlow *et al.*, 2010; Halperin *et al.*, 2010).

As its name indicates, fHbp binds human factor H (fH), a key negative regulator of the alternative complement pathway. Binding fH enables the meningococcus to evade killing by human complement, and thereby enhances its survival *in vivo* (Madico *et al.*, 2006). However, the expression of fHbp varies several fold among invasive isolates, affecting its potential as a target for bactericidal antibodies. This has implications both for fHbp as a target antigen for vaccination and for the evaluation of anti-fHbp serological responses *in vitro* (Giuliani *et al.*, 2010) using tests such as the serum bactericidal assay (SBA) or the meningococcal antigen typing system (MATS) (Donnelly *et al.*, 2010). Based on microarray data it has been suggested that the *fHbp* gene is a member of the FNR (fumarate and nitrate reductase) regulon and that its expression is therefore subject to oxygen availability (Bartolini *et al.*, 2006). Under anaerobic conditions, FNR is present as a dimer containing an iron–sulphur cluster. The FNR dimer binds to consensus sequences in the promoters of various genes and enhances transcription. In the presence of oxygen, the iron–sulphur cluster is degraded and the dimer loses its ability to bind DNA, resulting in reduced transcription of upregulated genes (Edwards *et al.*, 2010). Recent evidence shows that fHbp is expressed from two independent transcripts, one of which is under the control of a promoter that responds to oxygen limitation in an FNR-dependent manner (Oriente *et al.*, 2010). In addition, upstream of the *fHbp* promoter sequence in the Z2491 genome there is a nucleotide sequence that has similarities to a fragment of an insertion element (IE). This nucleotide sequence is not present upstream of the *fHbp* gene in other published genomes, but whether it affects the expression of *fHbp* is currently unknown (Oriente *et al.*, 2010). Although the role of fHbp in binding complement factor H is well established, other possible functions of this protein have been identified. For example, fHbp has been shown to be important for survival of meningococcal cells in the presence of the antimicrobial peptide LL-37 (Seib *et al.*, 2009) and to bind ferric enterobactin *in vitro* (Rappuoli, 2010). Initial characterization of fHbp also showed sequence and structural similarity to the meningococcal transferrin binding proteins (Cantini *et al.*, 2006; Masignani *et al.*, 2003).

In this study, the transcription levels of *fHbp* were compared across 90 meningococcal isolates grown in iron-restricted and iron-sufficient media. Isolates used were from the Multi-Locus Sequence Typing (MLST) *Neisseria* reference set (Maiden *et al.*, 1998), including both disease and carriage isolates collected between 1937 and 1996 from a variety of clonal complexes (ccs) and geographical locations. Furthermore, the availability of the whole-genome sequences allowed the *fHbp* gene and upstream sequence to be compared among the isolates (H. B. Bratcher and others, unpublished results). The study revealed differences in the transcription of *fHbp* among different ccs of the meningococcus and raised further questions about the evolved functions of this protein.

## Methods

### 

#### Isolates.

*N. meningitidis* isolates investigated were from the published MLST reference set (Maiden *et al.*, 1998). Information on all isolates is listed in Supplementary Table S1.

For all 107 isolates, sequences of *fHbp*, *cbbA* and the intergenic (IG) region between these genes were from the BIGSdb (Jolley & Maiden, 2010), hosted in the Department of Zoology at the University of Oxford, using the blast function. Sequences were aligned and compared using dnastar Lasergene (v. 8.0.2) software.

MLST allelic profile data were also downloaded from the BIGS database (Jolley & Maiden, 2010). A majority-rule consensus tree depicting the phylogeny of the isolates was obtained from Xavier Didelot (University of Oxford) (Didelot *et al.*, 2009) and edited using mega version 5 software.

#### Isolation of total RNA.

Nine isolates from the MLST reference collection were not available for RNA extraction. RNA was prepared from the remaining 98 isolates. Bacteria were resuspended from overnight growth on blood agar plates (Oxoid) into Mueller–Hinton broth (MH; Oxoid) or MH depleted of available iron by the addition of 50 µM deferoxamine mesylate (DFAM) salts (Sigma Aldrich) as a chelator. After resuspension to OD_650_ 0.18–0.22, meningococci were grown at 37 °C with 150 r.p.m. rotational shaking for 5 h. To protect the RNA from enzymic degradation the culture was mixed with RNAprotect Bacteria reagent (Qiagen), in a ratio of one volume culture to two volumes reagent, and incubated at room temperature for 5 min. Bacterial cells were harvested by centrifugation at 9600 ***g*** for 10 min and the resulting cell pellets were frozen overnight at −80 °C. Total RNA was extracted from the cell pellets using an RNeasy Mini kit, and DNA was removed by on-column RNase-free DNase digestion (Qiagen). Total RNA was eluted in 50 µl RNase-free water.

#### Quantitative real-time PCR (qRT-PCR) assay.

Custom primers (Thermo Fisher) and probes (TIBMolbiol) used for gene expression assays are listed below. Fluorescent markers used were 6JOE (6-carboxy-4′,5′-dichloro-2′,7′-dimethoxyfluorescein) and 6FAM (fluorescein). BlackBerry Quenchers (BBQ) were used on all probes.

Primers used for *gdh* were gdh350F (5′-TCGCCATTAAAGCCGAAATC-3′), gdh417R (5′-CTTGCCGGTACGCAGGTAGA-3′) and gdh374T (5′-6JOE-ACGAACGCTGGAAGGGCGTTC-BBQ-3′). Primers used for *fHbp* (assay 1) were fHbp117F (5′-CGTCAGGAAAAACGAGAAACTGA-3′), fHbp186R (5′-GCTGTCGCCGTTTCCATAAG-3′) and fHbp142P (5′-6FAM-CTGGCGGCACAAGGTGCGG-BBQ-3′). Primers used for *fHbp* peptide 1 (assay 2) were fHbp407F (5′-GCGAACATACATCTTTTGACAAGCT-3′), fHbp468R (5′-CGCCGTCCCGCGATA-3′) and fHbp435P (5′-6FAM-CGAAGGCGGCAGGGCGAC-BBQ-3′). Primers used for *cbbA* were nmb1869F (5′-GGAGACACAAATGGCACTCGTA-3′), nmb1869R (5′-GGCAGGCCGTAGCTGTTTT-3′) and nmb1869P (5′-6FAM-CATGCGCCAACTGCTTGATCATGC-BBQ-3′). Amplicon sizes were as follows: *gdh*, 69 bp; *fHbp* assay 1, 70 bp; *fHbp* assay 2, 62 bp; *cbbA* 72 bp.

Primer and probe sequences were determined using Primer Express 2.0 software (Applied Biosystems). mRNA from the majority of isolates (82 isolates in total; Supplementary Table S1) was detected by a single assay (assay 1). However, due to sequence variation, *fHbp* peptide 1 (variant 1.1 according to the MATS typing scheme) was amplified with a *fHbp* variant-specific primer/probe set (assay 2, eight isolates). The *fHbp*s from eight isolates were not amplified by either set of primers and probes, and were thus omitted from further investigation (Supplementary Table S1).

RT-PCR assays were completed in MicroAmp Fast Optical 96-Well Reaction Plates sealed with MicroAmp Optical Adhesive Film (both Applied Biosystems). Expression of the housekeeping gene *gdh* was used as an internal control in each well. For both *fHbp* and *cbbA*, reactions were mixed to a final volume of 23 µl with reagents at the following final concentrations: 1× TaqMan RT Enzyme Mix, 1× TaqMan RT-PCR Mix (both from the TaqMan RNA-to-CT 1-Step kit, Applied Biosystems), 300 nM each target and control primers, 200 nM target probe and 200 nM *gdh* probe. A 2 µl volume of total meningococcal RNA was added to each reaction. A negative RNA-free control and a standard positive control sample of H44/76 total RNA (following growth in MH) were run on each plate. All reactions were run in triplicate.

RT-PCR was completed using an Applied Biosystems 7500 Fast RT-PCR system with the following thermocycling conditions: 48 °C for 15 min; 95 °C for 10 min; 40 cycles of 95 °C for 15 s, 60 °C for 60 s. Fluorescence was recorded at the end of each extension step. Relative quantity (RQ) values were calculated with the ${\backslash rm2}^{–\Delta \Delta {\mathrm{\backslash it C}}_{\text{t}}}$ method by the Applied Biosystems 7500 Fast System Sequence Detection software. The *gdh* reaction was used as an endogenous control. The H44/76 positive control sample was set as the ‘calibrator sample’ on each plate, to which all RQ values were normalized.

#### Detection of bicistronic transcript.

To detect transcription of the upstream IG region, the primers IGF (5′-ACAAAATGCCGTCTGAAC-3′) and IGR (5′-CATCAATGAGGCAGGTCA-3′) were used in an RT-PCR assay with total RNA as described above using the SYBR Green RNA-to-CT 1-Step kit (Applied Biosystems). The *gdh* primers were used in a separate reaction for each RNA sample as a positive control. Thermocycling conditions were as described above, and the fluorescence released by the SYBR Green dye was recorded at the end of each extension step. Following amplification, PCR products were resolved at 100 V for 2 h on acrylamide TRIS-borate-EDTA (TBE) gels containing: 13 % (v/v) bis-acrylamide (29 : 1, Bio-Rad), 1× TBE, 0.16 % (w/v) ammonium persulfate (NBS Biologicals) and 0.08 % (v/v) *N*,*N*,*N*′,*N*′-tetramethylene-diamine (TEMED; Bio-Rad). Gels were incubated in 1× TBE containing 0.005 % (v/v) Safeview Nucleic Acid Stain (NBS Biologicals) for 20 min prior to detecting the bands under UV light. Successful amplification was indicated by the presence of bands corresponding to amplicons of 69 bp for both the IG region and *gdh*.

## Results

### Comparison of *fHbp* upstream regions

**Fig. 1.**
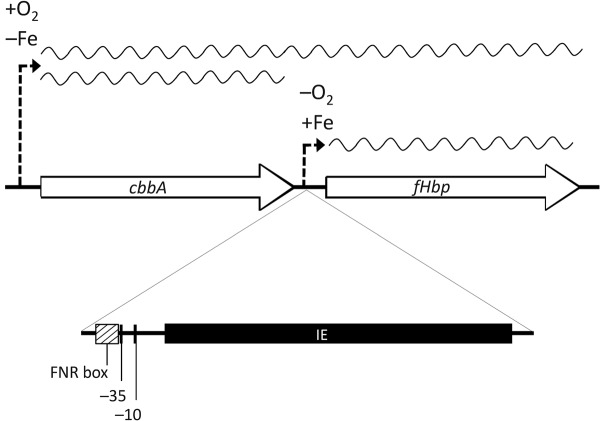
Genomic context of *cbbA* and *fHbp*, showing the position of the 181 bp IE relative to the *fHbp* coding region in the Z2491 genome sequence. The positions of the FNR-binding sequence (FNR box) and −35 and −10 regions (labelled), as determined elsewhere (Oriente *et al.*, 2010), are shown. The RNA transcripts (both monocistronic and bicistronic) and directions of oxygen regulation demonstrated in meningococcal strain MC58 are also shown.

**Fig. 2.**
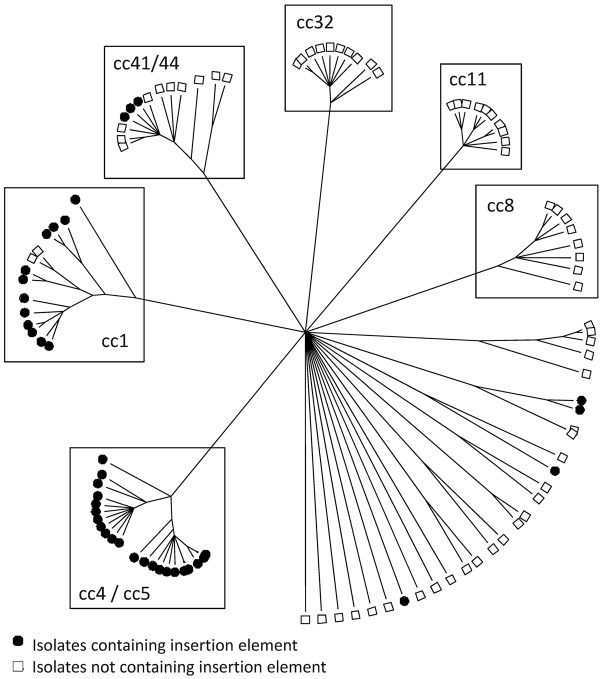
Distribution of the presence of the IE upstream of the *fHbp* gene across the ccs present in the isolate set investigated. The tree shows the majority-rules consensus relationships between all isolates when using 20 housekeeping gene fragments, as determined previously (Didelot *et al.*, 2009). Each terminal node on the tree corresponds to a single isolate. Isolates are shown as either containing the IE (•) or not containing the IE (□). ccs containing more than five isolates in the panel are boxed.

The nucleotide sequences of *fHbp* and its upstream region in all 107 MLST reference isolates were extracted from the BIGSdb (Jolley & Maiden, 2010) and aligned using dnastar Lasergene megalign (v. 8.0.2) software. In 64 of the isolates analysed the upstream region was highly conserved, with >87.6 % sequence identity. However, in 43 of the investigated isolates there was a 181 bp IE upstream of the coding region. This IE was observed in a single meningococcal isolate by Oriente *et al.* (2010), and was predicted to be positioned downstream of the −10 region (Oriente *et al.*, 2010) (Fig. 1). The IE was identified in all isolates belonging to sequence types (STs) 1, 4 and 5 (Fig. 2). Further scrutiny of the sequence data from the reference isolates in BIGSdb revealed that the IE was present at alternative positions among the different genomes rather than upstream of the *fHbp* gene, often occurring at multiple sites within the genome. The location of the IE varied among isolates, although many isolates contain the sequence between the convergent ORFs *mtrR* and *nmb1718*.

### Effect of iron availability on *fHbp* transcription levels

**Fig. 3.**
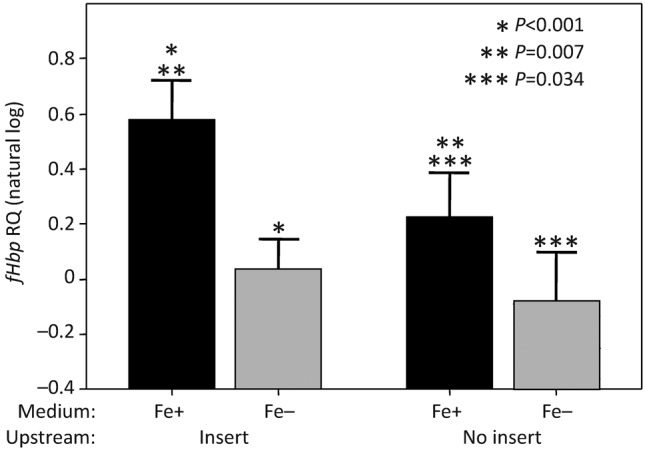
Comparison of the effect of iron availability on *fHbp* transcription levels in all 82 isolates tested using assay 1. Error bars, upper 95 % confidence intervals of the mean. Due to the use of different assays, isolates expressing fHbp variant 1.1 (eight isolates, all cc32) could not be directly compared with other isolates and were analysed separately. When grown in iron-restricted media (Fe−, grey bars), *fHbp* RQ values were significantly lower than when grown in iron-sufficient media (Fe+, black bars). This effect was highly significant in isolates containing the upstream insert (*P*<0.001), but the difference was reduced in isolates without the insert (*P* = 0.034).

To investigate the effect of iron on *fHbp* transcription, levels of *fHbp* mRNA extracted from isolates grown under iron-replete and -restricted conditions were determined using qRT-PCR. In the majority of isolates tested (assay 1, 82 isolates in total), levels of mRNA were significantly higher under iron-replete conditions than iron-restricted conditions (*P*<0.001, Fig. 3). This effect was more pronounced in isolates containing the IE upstream of the gene than in those without the IE in this position (*P* = 0.007, Fig. 3). In strains containing the IE the mean RQ natural log under iron-replete conditions was 0.578±0.139 versus 0.034±0.108 under iron-restricted conditions (*P*<0.001). In comparison, for strains not containing the IE, the mean RQ natural log under iron-replete conditions was 0.226±0.160, versus −0.076±0.72 under iron-restricted conditions (*P* = 0.034).

**Fig. 4.**
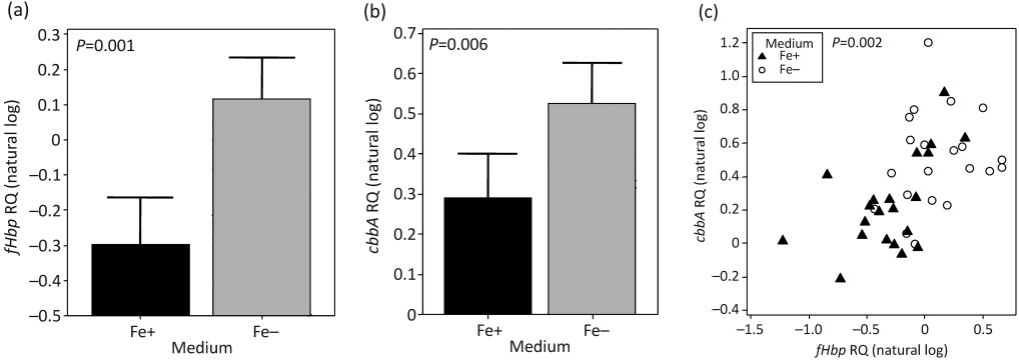
Comparison of the effect of iron availability on *fHbp* and *cbbA* transcription levels in all isolates belonging to cc32 (eight isolates in total, expressing fHbp variant 1.1). Cultures were grown in iron-restricted and iron-replete media on three separate occasions. Bars show the mean RQ values for all RNA extractions (Fe−, grey bars; Fe+, black bars); error bars, upper 95 % confidence intervals of the mean. (a) Effect of iron availability on *fHbp* RQ values. A single isolate, NG 080, was excluded from the analysis of *fHbp* as the RQ values for this isolate were very low in comparison with other cc32 isolates (mean RQ natural log −3.552 under iron-replete conditions, −3.827 under iron-restricted conditions). When grown in iron-restricted media, *fHbp* RQ values were found to be higher than when grown in iron-sufficient media. When tested by two-sample *t* test, excluding NG 080, the effect of iron on *fHbp* RQ values was found to be significant (*P* = 0.001). (b) Effect of iron availability on *cbbA* RQ values in all cc32 isolates. When grown in iron-restricted media, *cbbA* RQ values were found to be significantly higher than when grown in iron-sufficient media (*P* = 0.006). (c) Correlation between transcription of *fHbp* and *cbbA* in cc32 isolates. RQ values from Fe+ (▴) and Fe− (○) cultures. There was a significant correlation between *fHbp* RQ and *cbbA* RQ values of cc32 isolates (*P* = 0.002), with the exception of NG 080.

Conversely, most isolates belonging to cc32 produced significantly less *fHbp* mRNA under iron-replete conditions than iron-restricted conditions (mean RQ natural log = 0.299±0.164 under iron-replete conditions versus 0.166±0.142 under iron-restricted conditions, *P* = 0.001) (Fig. 4a). One cc32 isolate, NG 080, showed very low *fHbp* transcription levels, regardless of iron availability.

### Co-regulation of *fHbp* and *cbbA*

**Fig. 5.**
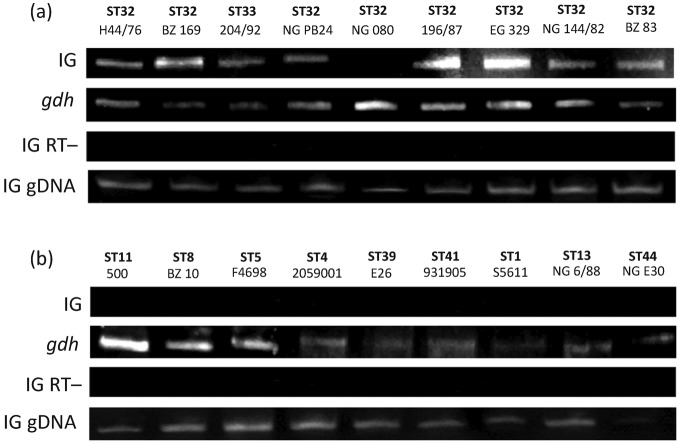
PCR products from amplification of cDNA of the IG region upstream of *fHbp*, with amplification of *gdh* (69 bp) as a positive control. Control reactions were also run in the absence of reverse transcriptase in order to detect contaminating DNA (IG RT−) and with genomic DNA to confirm that the appropriate sequence was present for primer binding (IG gDNA). The presence of a PCR product from amplification of the IG region (69 bp) suggests the expression of a bicistronic transcript. (a) Amplification from total RNA of the cc32 isolates in the reference isolates set; (b) amplification from total RNA of nine isolates from various ccs. Isolates F4698, 2059001, E26 and S5611 contained the IE.

In the meningococcal isolate MC58, belonging to cc32, fHbp is expressed as part of a bicistronic transcript from the upstream gene *cbbA* (Oriente *et al.*, 2010). To investigate this observation further, PCR was used to amplify the IG region between *cbbA* and *fHbp* from total RNA extracted from all eight cc32 isolates in the reference panel as well as from nine diverse isolates representing other ccs and *fHbp* alleles, including four isolates that contained the IE upstream of *fHbp*. SYBR Green dye was used to follow the amplification of cDNA during the RT-PCR, and PCR products were subsequently electrophoresed on an acrylamide gel to verify that PCR products of the expected size were present. The positive control template *gdh* was successfully amplified from all the RNA samples tested. The amplification of the IG region suggested that the ability to produce a bicistronic transcript was restricted to isolates of cc32: no isolates tested from other ccs produced a PCR product of the expected size (69 bp, Fig. 5). A single cc32 isolate in this set, NG 080, did not show the presence of the intergenic transcript.

To investigate whether the gene situated upstream of *fHbp*, *cbbA*, is also iron-regulated, the level of transcription following growth under iron-replete and -depleted conditions was measured using qRT-PCR. The cc32 isolates present in the isolate collection exhibited significant iron restriction of *cbbA* transcription [mean RQ natural log 0.237±0.127 under iron-replete conditions versus 0.499±0.130 under iron-restricted conditions, *P* = 0.006; Fig. 4(b)]. Isolates tested from other ccs showed iron-repressed transcription of *cbbA* similar to that of cc32 isolates (data not shown). For isolates in which expression of the intergenic transcript was detected, regression analysis showed a significant linear correlation between RQ values obtained for *fHbp* and *cbbA* [*P* = 0.002; Fig. 4(c)].

## Discussion

The fHbp of *N. meningitidis* is a component of two vaccines developed ostensibly to control serogroup B meningococcal disease (Donnelly *et al.*, 2010; Jiang *et al.*, 2010). The currently accepted correlate of protection used to evaluate such vaccine candidates is the ability to induce functional antibodies, as measured in an SBA (Borrow *et al.*, 2006). To evoke an effective bactericidal response against the meningococcus, it is critical that sufficient fHbp is expressed on the surface of the bacterium (Koeberling *et al.*, 2011), and there is evidence that the level of expression of the protein on the bacterial surface correlates with the susceptibility of target isolates to fHbp-specific antibodies in an SBA (Jiang *et al.*, 2010). The level of fHbp expression *in vitro* varies among meningococcal isolates. Furthermore, regulation of *fHbp* is likely to vary within the host, depending on levels of exogenous nutrients. Current evidence that oxygen availability plays a role in the regulation of *fHbp* expression, via the regulatory protein FNR, supports this assumption (Bartolini *et al.*, 2006; Oriente *et al.*, 2010). The findings of this and previous studies indicate that the regulation of *fHbp* is intricate and differs among ccs.

The availability of iron varies within the human host, and the meningococcus has evolved multiple mechanisms for the acquisition of iron from its host sources (Genco & Desai, 1996), which is reflected in the numerous meningococcal genes that have been shown to be iron-regulated (Grifantini *et al.*, 2003). In this study, qRT-PCR measurements of the effect of iron availability on levels of transcription of the *fHbp* gene in a diverse collection of meningococci showed that, for the majority of the isolates, growth in iron-depleted conditions resulted in significantly lower levels of *fHbp* transcription than growth in iron-replete media. In contrast, cc32 isolates showed significantly higher levels of *fHbp* transcription when grown under iron-restricted conditions. This observation suggests that different regulatory mechanisms act upon this gene depending on the genotype of the isolate investigated.

Earlier work (Oriente *et al.*, 2010) demonstrated that *fHbp* RNA in the cc32 isolate MC58 is present in two different transcripts: a transcript expressed from the *fHbp* promoter, and a bicistronic transcript also containing the upstream gene *cbbA* (Oriente *et al.*, 2010). Employing RT-PCR, the co-transcription of these genes was investigated across the collection of isolates, showing that the presence of the bicistronic transcript was confined to isolates belonging to cc32. Transcriptional termination in *Neisseria* is thought to occur frequently at Rho-independent terminators (Kingsford *et al.*, 2007), which are formed of palindromic loops in DNA secondary structure that prevent advancement of the polymerase, although the structure and stability of these loops is dependent on the DNA sequence present (Abe & Aiba, 1996). The sequences of the IG region between *cbbA* and *fHbp* were compared among all isolates, and several nucleotide polymorphisms were detected in cc32. This sequence variation could result in a shorter or less stable palindromic loop, allowing DNA polymerase to read through the putative transcriptional terminator in cc32 isolates.

We have shown that the control of *fHbp* transcription in cc32 isolates grown in iron-restricted MH media was dominated by the *cbbA* promoter. The *cbbA* gene encodes a fructose-1,6-bisphosphate aldolase, a metabolic enzyme involved in glycolysis. In this bicistronic transcriptional arrangement, transcription from the *fHbp* promoter itself appeared to be relatively unimportant. The only cc32 isolate in this study that did not transcribe the IG region, NG 080, also had low levels of *fHbp* transcription when compared with other isolates analysed. Furthermore, there was a good correlation between transcription levels of *cbbA* and *fHbp* when isolates were grown under iron-replete or -depleted conditions. This is consistent with earlier observations that removal of *cbbA* transcription in MC58 results in significantly reduced levels of *fHbp* transcription and protein expression (Oriente *et al.*, 2010).

It has been shown that transcription of *cbbA* is dependent on oxygen but independent of FNR. In contrast to the FNR-regulated *fHbp*, oxygen enhances the rate of transcription of *cbbA* (Oriente *et al.*, 2010). Consequently, under conditions of excess iron and excess oxygen, opposing regulatory mechanisms would be acting on transcription of both *cbbA* and *fHbp*. Iron and oxygen availability are intrinsically linked *in vivo*, as oxygen potential affects the strength of the association between iron and host iron-binding proteins (Bullen *et al.*, 1992). Iron is also known to be involved in the oxygen-dependent regulatory mechanism of FNR, as the presence of an iron–sulfur cluster within an FNR dimer is required for the interaction with DNA. In the presence of oxygen, this iron–sulfur cluster is displaced (Crack *et al.*, 2004). The relative strengths of iron- and oxygen-dependent regulation on both *fHbp* and *cbbA* warrant further investigation.

Finally, the observation of an AT-rich IE downstream of the predicted *fHbp* promoter region in the meningococcal isolate Z2491 has been extended. Comparison of the genome sequences of the 107 reference isolates identified the 181 bp IE at the same location in all ST1, ST4 and ST5 isolates, as well as its presence in alternative positions at least once in all 107 genome sequences. A blast search of this sequence demonstrated the presence of identical sequence in genomes of *Neisseria gonorrhoeae* isolates. The presence of multiple copies of identical sequence in non-coding regions of the meningococcal genomes is consistent with the hypothesis that this sequence is likely to be an IE (Oriente *et al.*, 2010); however, there is no significant homology with any sequence outside of these species and the sequence does not contain the DNA uptake sequence (Ambur *et al.*, 2007) that is normally associated with DNA IEs in *Neisseria* species. The presence of the IE upstream of the *fHbp* gene was associated with higher levels of gene transcription following growth in iron-replete media. The mechanism behind this difference is unknown, but this observation further highlights the complicated nature of transcriptional regulation of the *fHbp* gene and how it can vary among different meningococcal genotypes.

The results of the present study, combined with earlier observations (Bartolini *et al.*, 2006; Oriente *et al.*, 2010), suggest that *fHbp* transcription is regulated by both oxygen and iron availability. Assuming that levels of transcription reflect the levels of fHbp expression, this has implications for the use of biological assays, such as the SBA and MATS, in the evaluation of vaccine potency and coverage. For example, SBAs are usually carried out following growth of target isolates on the iron-rich medium blood agar (Maslanka *et al.*, 1997). These conditions enhance transcription of *fHbp* in most meningococcal isolates, while repressing transcription of *fHbp* specifically in target isolates belonging to cc32. To offer protection *in vivo*, antibodies would be required to bind to meningococci in an iron-deficient milieu (Perkins-Balding *et al.*, 2004). Therefore, the *in vitro* conditions used for SBAs may overestimate the protection offered by vaccine candidates for the majority of meningococcal isolates, while specifically underestimating protection against cc32 isolates, many of which express the fHbp variant 1.1, which is the variant included in the Novartis vaccine formulation (Jacobsson *et al.*, 2009).

Given the close relationship between the regulation of gene expression and the function of the corresponding gene product, the iron-dependent expression of *fHbp* also raises questions about the predicted role of this protein in meningococcal survival. Increased sensitivity to killing in whole human blood and human sera has been demonstrated in *fHbp*-knockout mutants (Seib *et al.*, 2010). However, much of this work has been carried out in isolates MC58 and H44/76, both of which are members of cc32. The varying regulation of *fHbp* in isolates from other ccs, seen in this study, suggests that the protein may have an alternative function in these bacteria. This would be consistent with data showing that the ability to bind fH varies widely across different fHbp variants (Seib *et al.*, 2011) and that, in certain isolates, fHbp expression can be removed without any effect on the serum resistance of those isolates (Welsch *et al.*, 2008).

The increasing evidence of an association between antigen expression levels and bactericidal killing (Donnelly *et al.*, 2010) highlights the importance of understanding antigen regulation at a genetic level. Meningococci are known to be highly diverse and adaptable organisms (Feavers, 2000), and it is perhaps unsurprising that regulation of certain ORFs differs among isolates. It is important that this diversity in antigen regulation is investigated and ultimately incorporated into estimates of potential vaccine coverage.
